# Supplementary material for: Mapping the evolution of fertility support policies in China: A content and instrumental analysis
Source: PLoS One. 2025 Oct 9;20(10):e0332137. doi: 10.1371/journal.pone.0332137 (PMC12510515; doi:10.1371/journal.pone.0332137)
Supplement: S1 Appendix — (ZIP) [file pone.0332137.s001.zip › S1 Appendix. 226 original policy documents/28-国家卫生计生委、国家发展改革委、教育部等关于加强生育全程基本医疗保健服务的若干意见(FBM-CLI-4-282872).doc]

国家卫生计生委、国家发展改革委、教育部等关于加强生育全程基本医疗保健服务的若干意见

**发布部门：** [国家卫生和计划生育委员会(已撤销)](https://www.pkulaw.com/law/chl?Aggs.IssueDepartment=60319) [国家发展和改革委员会(含原国家发展计划委员会、原国家计划委员会)](https://www.pkulaw.com/law/chl?Aggs.IssueDepartment=60301) [教育部](https://www.pkulaw.com/law/chl?Aggs.IssueDepartment=60203) [机构沿革](javascript:void(0))

**发文字号：**国卫妇幼发〔2016〕53号

**发布日期：**2016.10.14

**实施日期：**2016.10.14

**时效性：** [现行有效](https://www.pkulaw.com/law/chl?Aggs.TimelinessDic=01)

**效力级别：** [部门规范性文件](https://www.pkulaw.com/law/chl?Aggs.EffectivenessDic=XE0302)

**法规类别：** [卫生综合规定](https://www.pkulaw.com/law/chl?Aggs.Category=09601)

国家卫生计生委、国家发展改革委、教育部、财政部、人力资源社会保障部关于加强生育全程基本医疗保健服务的若干意见
（国卫妇幼发〔2016〕53号）

各省、自治区、直辖市及新疆生产建设兵团卫生计生委（卫生局）、发展改革委、教育厅（局）、财政（务）厅（局）、人力资源社会保障厅（局）：
　　为贯彻落实《中共中央 [国务院关于实施全面两孩政策改革完善计划生育服务管理的决定](https://www.pkulaw.com/chl/8564756bae805261bdfb.html)》（中发〔2015〕40号）和“十三五”时期国民经济和社会发展规划纲要精神，进一步深化医药卫生体制改革，加强生育全程基本医疗保健服务，提升妇女儿童健康水平，保障全面两孩政策顺利实施，现提出以下意见。
　　**一、**优化妇幼健康服务资源配置
　　（一）尽快调整扩增妇幼健康服务资源。省、地市、县各级要迅速摸清区域内现有产科服务资源底数，预估新增生育峰值，合理测算需求缺口。根据全面两孩政策实施后群众新增需求，以“调整存量、做优增量、补齐短板、提升能力”为原则，争取达到每千分娩量产科床位数17张。充分利用各级各类医疗机构现有资源，通过科室间、科室内床位调整等方式，尽快扩增产科床位，缓解部分地区“一床难求”的突出问题。生育服务需求大的重点地区可探索分级建档制度，合理分流，引导孕妇根据风险评估结果合理选择建档机构；设立孕妇建档服务中心，协调解决孕妇建档问题，让每位孕妇都能得到及时、安全、有效的孕产期保健服务。加快实施妇幼健康和计划生育服务保障工程，加强省、地市、县三级妇幼保健机构建设，在县级医院建设项目中着力提高产科服务能力，新增产科床位8.9万张，力争在“十三五”前期解决妇幼健康服务资源总体不足和结构性短缺的供需矛盾。
　　（二）积极推进优质妇幼健康服务资源下沉。通过组建妇幼健康服务联合体、远程医疗、对口支援等方式，促进优质妇幼健康服务资源下沉，提高基层医疗卫生机构服务能力。鼓励提供妇幼健康服务的省、地市级医疗机构与县级医疗机构、县级医疗机构与基层医疗机构纵向联合，联合体内部可通过项目合作、联合病房、学科帮扶、远程会诊等形式加强合作，形成分工协作、上下联动的工作机制，共建共享，提升整体服务能力和效能。引导区域间提供妇幼健康服务的医疗机构围绕学科建设互帮互助、优势互补，促进区域间妇幼健康服务能力均衡发展。
　　（三）着力提升孕产妇和新生儿危急重症救治能力。省、地市、县要依托产科儿科实力突出和综合救治能力较强的医疗机构，加快孕产妇和新生儿危急重症救治能力建设。完善服务设施，加强人员配备，健全运行机制，畅通危急重症救治绿色通道，确保2018年底前建成分级负责、上下联动、应对有序、运转高效的孕产妇和新生儿危急重症急救、会诊、转诊网络。
　　（四）全面加强急需紧缺专业人才培养使用。加快产科医师、助产士人才培养，力争“十三五”时期增加产科医生和助产士14万名。在有条件的高校探索开设大学本科助产相关专业，加强招生培养工作。实施助产士转岗培训计划，加强助产士专业方向的继续医学教育，完善助产士评价标准。在基层卫生人员培训中加强妇幼健康服务知识技能培训。组织符合条件的妇幼保健机构积极承担住院医师规范化培训工作任务，加强妇幼保健专业内容培训，打造一支临床医学专业基础扎实、公共卫生视角宽广的复合型人才队伍。完善医疗卫生机构绩效评价和人才激励机制，在绩效工资内部分配等方面对产科医师、助产士、护士等给予倾斜，改善医护人员待遇，增加岗位吸引力。

　　**二、**加强生育全程优质服务
　　（五）关口前移，加强生育前咨询与服务。开展助产技术服务的医疗机构（以下简称助产机构）要积极设立生育服务咨询室，为有生育需求的夫妇提供有针对性的综合服务，做好孕前优生健康检查，加强健康咨询服务，规范提供生育力评估及科学备孕指导、妊娠风险提示等服务。帮助再生育夫妇终止长效避孕措施，向生育困难的夫妇规范提供中医药调理、药物治疗、妇产科常规手术、人类辅助生殖技术服务等不孕症综合治疗。
　　（六）系统整合，做好生育全程服务。在总结试点经验基础上，在全国推广使用统一的母子健康手册，整合孕前保健、孕期保健、住院分娩、儿童保健、儿童预防接种和计划生育服务内容，为妇女儿童提供系统、规范的优生优育全程服务，打造“一条龙”服务链。开展生育全程医疗保健服务，涵盖婚前、孕前、孕产、产后、儿童等5个时期，主要包括婚前保健、孕前保健、早孕建册、产前检查、产前筛查与诊断、住院分娩、产后访视、预防疾病母婴传播、新生儿疾病筛查、儿童健康管理、儿童营养改善、预防接种、计划生育技术服务等13项服务。落实生育登记服务制度。做好流动孕产妇和儿童跨地区利用服务的接续。
　　（七）抓住关键，强化妊娠风险评估管理。引导孕产妇就近、有序接受孕产期保健服务。医务人员要认真询问既往生育史、难产史、避孕史，详细进行体格检查。在监测评估基础上，引导高龄孕产妇到二级及以上医疗机构进行孕产期保健和分娩。要对孕产妇尤其是高龄孕产妇进行妊娠风险评估，筛查危险因素，识别高危孕产妇，严格实行高危孕产妇专案管理，密切监测、治疗妊娠合并症和并发症。完善孕产妇和新生儿危急重症转诊、救治网络，保障危重孕产妇和新生儿及时转运救治，确保有效衔接和绿色通道畅通。
　　（八）突出重点，推进出生缺陷综合防治。建立覆盖城乡居民，涵盖孕前、孕期、新生儿各阶段的出生缺陷防治服务制度。推广出生缺陷一级防治措施，加强宣传和健康教育，倡导婚前医学检查，推进免费孕前优生健康检查，为孕前及孕早期妇女增补叶酸。落实出生缺陷二级防治措施，不断扩大产前筛查人群覆盖面，做好后续产前诊断服务，提高孕期出生缺陷发现率和干预率。加强出生缺陷三级防治措施，提高新生儿疾病筛查率，扩大新生儿疾病筛查病种，促进患儿早诊早治。完善基因检测技术临床应用管理，推进基因检测技术应用示范中心建设，推动基因检测技术在出生缺陷综合防治中的科学应用。做好唐氏综合征、耳聋、地中海贫血、先天性心脏病等疾病的出生缺陷综合防控工作，努力减少出生缺陷发生，提高出生人口素质。
　　（九）中西医并重，促进妇幼保健与中医药融合服务。在提供妇幼健康服务的医疗机构积极推广应用中医适宜技术和方法，开展中成药合理使用培训。加强妇幼保健机构中医科室建设，提升妇幼保健机构中医药服务能力。加强妇女儿童疾病诊疗中西医临床协作，提高疑难病、危急重症诊疗水平。充分发挥中医药“治未病”优势，扩大中医药在孕育调养、产后康复、儿童保健等方面应用，努力减少妊娠并发症以及儿童常见病、多发病的发生。

　　**三、**完善妇幼健康服务模式
　　（十）推进防治结合服务模式。以保健为中心，保健与临床相结合，面向妇女儿童提供防治结合的健康管理服务。建立有利于防治结合的妇幼保健机构运行新机制，为妇女儿童提供预防保健服务和常见病诊疗服务。鼓励有条件的妇幼保健机构扩展强化产科、儿科等服务功能，在辖区妇幼健康服务体系中发挥骨干作用。以妇女儿童健康为中心，以妇幼保健机构评审为抓手，推动妇幼保健机构内部改革，规范设置业务部门，实现保健与临床业务实质融合。推进以妇幼保健机构为主体，与公立妇产医院、儿童医院有机结合，建立区域内防治结合的妇女儿童健康综合服务模式。
　　（十一）促进惠民政策有效衔接。促进生育过程中基本公共卫生服务项目、重大公共卫生服务项目、基本医疗保险、生育保险等相关政策措施有效衔接，创新工作机制，提高服务效能，增强群众获得感。完善公共卫生服务项目实施模式，对国家基本公共卫生服务项目中的孕产妇健康管理推广实施政府购买服务，提高服务质量和资金使用效率。加大对产前筛查等薄弱环节的支持力度，多渠道提供生育全过程的基本医疗保健服务。
　　（十二）推广便民利民服务举措。围绕妇女儿童医疗保健服务需求，优化诊室布局及诊疗流程，营造环境温馨、干净整洁、安全舒适的就诊环境。完善自助服务设备，提供便民服务设施，在儿科和儿童保健门诊积极设立母乳喂养室。全面开展孕产妇、儿童预约诊疗服务，逐步推广预约住院分娩。积极推行“互联网＋妇幼健康”服务模式，主动公布助产机构名单，有条件的地区要动态公布产科床位预约情况，引导群众有序就诊。广泛提供在线预约诊疗、候诊提醒、缴费支付、诊疗报告查询等便捷服务，切实改善群众就诊体验。
　　（十三）推动科技创新和成果转化。发挥科技创新在妇幼健康服务中的引领作用，打造妇幼健康高新技术平台，针对主要健康危险因素开展重大科技攻关。加强基础研究和科技创新，开发推广妇幼保健、生殖健康、避孕节育和优生优育新技术新产品，促进成果转化和技术进步。加强妇产科学、儿科学、妇幼保健学、遗传学、生殖医学等重点学科和国家级重点研究基地建设，加快适宜技术推广，使科技创新助力妇幼健康事业发展。创新母婴保健技术评估机制，规范新技术向临床应用转化。

　　**四、**落实政策保障措施
　　（十四）健全妇幼健康服务体系。进一步完善以基层医疗卫生机构为基础，妇幼保健机构、计划生育技术服务机构及妇女儿童专科医院为主体，大中型综合医院、相关科研教学机构为技术支撑，民营医院为补充的妇幼健康服务体系。推进妇幼保健和计划生育技术服务机构深度整合，省、地市、县均设置1所政府举办、标准化的妇幼保健机构。加强各级综合医院产科专科建设，提高产科疑难病症诊治能力。开展基层医疗卫生机构妇幼保健和计划生育服务科室的规范化建设，发挥基层医疗卫生机构在基本妇幼健康服务中的作用。鼓励和引导社会力量举办高水平、规模化的非营利性妇女儿童医院，满足人民群众多层次、多样化医疗服务需求。
　　在推进改革过程中，要保持妇幼保健机构稳定，坚守妇幼健康公益性服务的主阵地，设区的地（市）和县（区）级妇幼保健机构的变动应征求省级卫生计生行政部门的意见。不得以租赁、买卖等形式改变妇幼保健机构所有权性质；对造成国有资产流失的，依法追究相关责任人责任。
　　（十五）完善运行机制。结合深化医药卫生体制改革要求，逐步建立以成本和收入结构变化为基础的价格动态调整机制，合理调整产科、儿科医疗服务价格，重点提高体现医务人员技术劳务价值的服务价格，加快新增医疗服务价格项目受理审核。鼓励妇幼保健机构通过提供优质的预防保健和基本医疗服务获得合理收入，建立科学合理的绩效考核和激励机制。
　　（十六）强化监督管理。强化母婴保健技术监督执法，严格机构、人员准入，规范与妇幼健康相关的医疗保健服务。以产前诊断技术、助产技术和人类辅助生殖技术为重点，建立随机抽查制度。督促医疗机构落实医疗质量安全核心制度，健全医疗质量管理与控制体系，严格规范诊疗服务行为。严格控制剖宫产率，积极倡导母乳喂养，严格禁止非医学需要的胎儿性别鉴定和选择性别的人工终止妊娠。将妇幼健康信息系统纳入区域卫生计生信息规划，加强信息化建设与管理，加强妇幼健康信息安全保护。做好重点地区妇幼健康服务监测，建立母婴安全数据定期报送制度，加强工作指导针对性。
　　（十七）加强宣传与社会动员。做好政策解读与宣传，及时回应社会关注的热点问题，合理引导群众预期，提高人民群众对惠民政策的知晓率，提高群众自我保健意识和技能。大力宣传扎根基层、情系百姓的妇幼健康工作者，激励队伍、鼓舞士气，提高医务人员的积极性和能动性。广泛动员相关社会组织共同做好妇幼健康工作，重视发挥协会、学会作用，积极开展学术交流活动，协助做好宣传教育、咨询服务、优生优育指导等工作。
　　各地要高度重视，强化落实责任，将加强生育全程基本医疗保健服务摆在突出位置，纳入健康中国建设和实施全面两孩政策的总体部署，精心组织实施，强化督导检查，确保各项措施全面落实到位。

国家卫生计生委
国家发展改革委
教育部
财政部
人力资源社会保障部
2016年10月14日

© 北大法宝：（[www.pkulaw.com](https://www.pkulaw.com/)) 专业提供法律信息、法学知识和法律软件领域各类解决方案。北大法宝为您提供丰富的参考资料，正式引用法规条文时请与标准文本核对。
欢迎查看所有[产品和服务](https://www.pkulaw.com/helps/2.html)。法宝快讯：[如何快速找到您需要的检索结果？](https://www.pkulaw.com/helps/70.html)[法宝](https://www.pkulaw.com/helps/69.html)V6有何新特色？

扫描二维码阅读原文

**原文链接：**<https://www.pkulaw.com/chl/638b38583247f606bdfb.html>
